# Supplementary figures and images for: Bi-Level ventilation decreases pulmonary shunt and modulates neuroinflammation in a cardiopulmonary resuscitation model
Source: PeerJ. 2020 Apr 29;8:e9072. doi: 10.7717/peerj.9072 (PMC7195831; doi:10.7717/peerj.9072)

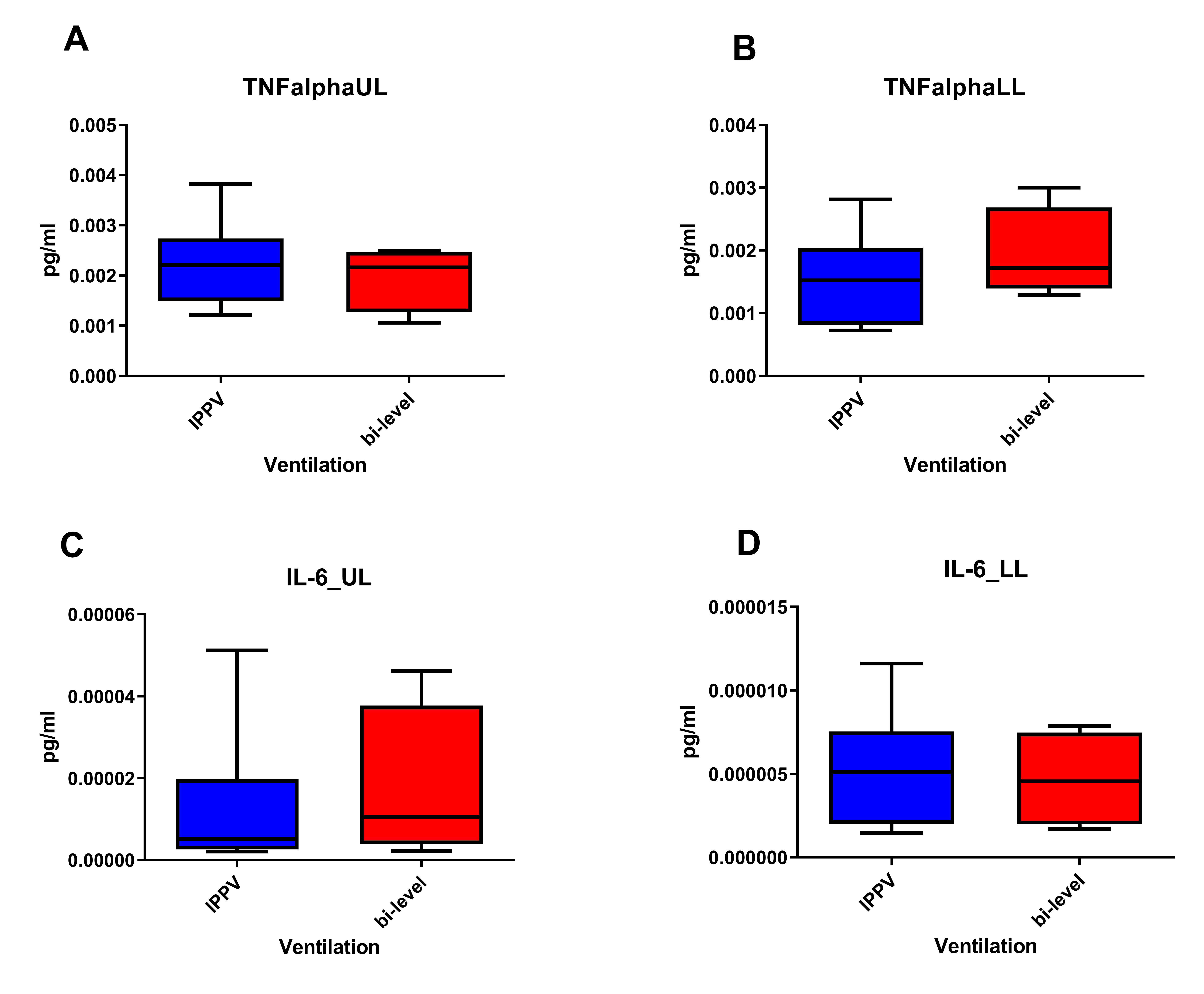

Supplement: Figure S1 — Analogous to Fig. 3, pulmonary tissue inflammation data is presented. No significant increase in inflammation could be detected in any lung segment. [file peerj-08-9072-s005.png]
